# Supplementary material for: Unmasking a gap: A new oligoneuriid fossil (Ephemeroptera: Insecta) from the Crato Formation (upper Aptian), Araripe Basin, NE Brazil, with comments on Colocrus McCafferty
Source: PLoS One. 2020 Oct 28;15(10):e0240365. doi: 10.1371/journal.pone.0240365 (PMC7592730; doi:10.1371/journal.pone.0240365)
Supplement: S1 Appendix — Morphological characters and their states coded for this phylogenetic analysis (from Massariol et al., [4], except where noticed). (DOCX) [file pone.0240365.s001.docx]

**S1 Appendix. Morphological characters.** Morphological characters and their states coded for this phylogenetic analysis (from Massariol *et al*., 2019, except where noticed).

1. Antenna, position: (0) dorsal; (1) frontal
2. Antenna, apical antennomeres, processes: (0) absent, (1) present
3. Anterior projection: (0) absent, (1) present
4. Eye, side, indent: (0) absent, (1) present
5. Eye, side, indent, amount: (0) one, (1) two
6. Labrum, anterior margin, spines: (0) absent, (1) present
7. Maxilla, apical angle, shape: (0) acute, (1) broad
8. Maxilla, maxillary palp, second segment length in relation to first: (0) subequal, (1) much longer
9. Labium, accessory gills: (0) absent, (1) present
10. Epimera and episterna, lateral margin extension: (0) not projected laterally, (1) projected laterally
11. Epimera and episterna, projection, shape: (0) rounded, (1) acute
12. Mid and hind coxae, shape: (0) cylindrical, (1) flattened
13. Mid and hind coxae, dorsal projection: (0) absent, (1) present
14. Fore femur, outer margin, fine, long, simple setae: (0) absent, (1) present
15. Fore femur, outer margin, fine, long, simple setae, position: (0) up to half the length, (1) exceeding
16. Mid and hind femora, outer margin, fine, long, simple setae: (0) absent, (1) present
17. Mid and hind femora, outer margin, fine, long, simple setae, position:(0) up to half the length, (1) exceeding
18. Mid and hind femora, inner margin, setae, protuberances at base: (0) absent, (1) present
19. Mid and hind femora, inner margin, setae, protuberances at base, development: (0) barely, (1) strongly
20. Fore tibia and tarsus, fusion: (0) fused, (1) not fused
21. Fore tibia, anterior surface, row of setae: (0) absent, (1) present
22. Fore tibia, anterior surface, row of setae, position: (0) restricted to apex, (1) along entire length
23. Mid and hind tibiae, inner margin, long, simple setae: (0) absent, (1) present
24. Mid and hind tibiae, inner margin, spine-like setae: (0) absent, (1) present
25. Mid and hind tarsal claw, shape: (0) slender, (1) robust
26. Abdominal terga, posterior margin, spines: 0) absent, (1) present
27. Abdominal terga, lateral margins, setae: (0) absent, (1) present
28. Abdominal terga, lateral margins, setae, type: (0) simple, (1) spine-like
29. Abdominal sterna, tuft of long setae: (0) absent, (1) present
30. Abdomen, posterolateral projection, segment distribution: (0) I-IV to IX, (1) VIII to IX
31. Abdominal sterna, segment I, finger-like process: (0) absent, (1) present
32. Gill I, insertion: (0) dorsal, (1) ventral
33. Gill I, outer lamella: (0) absent, (1) present
34. Gill I, outer lamella, development: (0) vestigial, (1) well developed
35. Gill IV, outer lamella, symmetricity: (0) symmetric, (1) asymmetric
36. Gill IV, outer lamella, dorsal surface and margins, spines: (0) absent, (1) present
37. Gill IV, outer lamella, length in relation to width: (0) about 5x, (1) about 2x
38. Gill IV, outer lamella, inner margin, long simple-setae: (0) absent, (1) present
39. Gill IV, inner lamella: (0) absent, (1) present
40. Gill IV, inner lamella, development in relation to outer lamella: (0) poor-developed (reaching half of total length of outer lamella), (1) well-developed (same length or longer than outer lamella)
41. Terminal filament: (0) absent, (1) present
42. Terminal filament, length in relation to length of cerci: (0) shorter (reaching half of length of cerci), (1) reaching at least 3/4 of length of cercus
43. Terminal filament, setae distribution: (0) almost along entire length, (1) along entire length, except in the basal 1/3
44. Caudal filaments, setae, amount: (0) abundant (covered with primary swimming setae in most segments), (1) rare (setae absent in most segment, only a few segments with scarce setae)
45. Caudal filaments, spine-like setae: (0) absent, (1) present
46. Male eye, continuity: (0) contiguous, (1) not contiguous
47. Plumidia: (0) absent, (1) present
48. Plumidia, length in relation to metanotum: (0) short (not reaching median length of abdominal segment I), (1) long (reaching median length of abdominal segment I)
49. Middle leg, coxae development: (0) not developed (around 1/5 of length of forefemur), (1) developed (at least 1/2 of forefemur)
50. Tarsal claws, shape: (0) at least one acute, (1) both rounded
51. Forewing, vein RP2: (0) absent, (1) present
52. Forewing vein RP2, origin in relation to length of forewing: (0) before basal third, (1) after basal third
53. Forewing, vein RP2, orientation in relation to RA: (0) parallel, (1) divergent
54. Forewing, IRP vein: (0) absent, (1) present
55. Forewing, longitudinal veins, gemination: (0) absent, (1) present
56. Forewing, vein IMP: (0) absent, (1) present
57. Forewing, vein IMP, translucency: (0) complete, (1) spectral
58. Forewing, vein MA, origin of bifurcation in relation to length of wing: (0) after basal third, (1) next to base
59. Forewing, vein RP, origin of bifurcation in relation to length of wing: (0) after basal quarter, (1) next to base
60. Forewing, complete crossveins present between veins, distribution: (0) all sectors, (1) restrict to 2 costal, subcostal, radial or median anterior (MA) sectors
61. Forewing, spectral crossveins: (0) absent, (1) present
62. Forewing, cubito-anal sector: (0) developed, (1) reduced
63. Forceps: (0) absent, (1) present 2
64. Forceps, pedestal of gonostyli: (0) absent, (1) present 1
65. Forceps, apical segments, division: (0) single, (1) divided
66. Forceps, apical segments, amount: (0) two, (1) three or more
67. Styliger plate, posterior margin, paired projection: (0) absent, (1) present
68. Penis, inner and outer lobe, fusion: (0) fused, (1) not fused
69. Penis, inner and outer lobe, articulation: (0) articulated, (1) free
70. Penis lobe, inner lobe: (0) membranous, (1) semi-membranous 4
71. Penis lobe, proximal sclerotized process: (0) absent, (1) present 1
72. Penis lobe, proximal sclerotized process, shape: (0) pointed, (1) saddle 1
73. Female, abdomen, posterolateral projection in the segment IX: (0) absent, (1) present
74. Forewing, cubital intercalaries: (0) present, (1) absent **(new)**
75. Forewing, vein IMA: (0) present, (1) absent **(new)**
76. Forewing, longitudinal veins, gemination (0) incomplete, (1) complete **(new)**
