# Supplementary material for: Unmasking a gap: A new oligoneuriid fossil (Ephemeroptera: Insecta) from the Crato Formation (upper Aptian), Araripe Basin, NE Brazil, with comments on Colocrus McCafferty
Source: PLoS One. 2020 Oct 28;15(10):e0240365. doi: 10.1371/journal.pone.0240365 (PMC7592730; doi:10.1371/journal.pone.0240365)
Supplement: S2 Appendix — Matrix of morphological characters and states used for the phylogenetic analyses of Oligoneuriidae. Ready for use in TNT format. (DOCX) [file pone.0240365.s002.docx]

**S2 Appendix. Matrix of characters.** Matrix of morphological characters and states used for the phylogenetic analyses of Oligoneuriidae. Ready for use in TNT format.

xread

76 23

Murphyella_needhami

0010?01000?1111110?10?01110?0?0011??1?1111?1110?00110101000000111000??0?100?

Isonychia

100??01010?1010100?111011011?000????????0??1100?00110101000000111000??0?000?

Rhithrogena

100??01000?1011110?10?01110??100111110111000000?0?111101000000111000??0??10?

†Incogemina_nubila

?????????????????????????????????????????????1????111111001000???????????000

†Colocrus_indivicum

??????????????????????????11?0?011??1???????????????????????????????????????

Chromarcys_magnifica

00111001111110?110?10?01101000001101101110100???01111101001000111100??0??00?

Oligoneuria_amazonica

011110011111110111010?0110100001100010111100011001101111111101100?11000?1111

Oligoneuria_mitra

011110011111110111010?0110100001100010111100011001101111111111100?11000?1111

Oligoneuria_anatina

011110011111111111110?1110100001110010111110011001101111111111101011000?1111

Lachlania_aldinae

01110001111110?111010?0110100001110010110??10???????????????????????????0111

Lachlania_alcidesi

01110001111110?111010?0110100001110010110??1011001111111011101100?11010?0111

Lachlania_saskatchewanensis

011??0011111?0?0???10?0110??0001110?10??0??0?1??01111111011101100?11010??111

Spaniophlebia

001110011111111111110?1110100001??0?10110??1011001101111011111100?01010?1111

Fittkauneuria

1010?00110?110?100?10?01100?01010?11110?10000111011011110111010???01??0??111

Homoeoneuria_watu

1010?10110?0011110?0??10000?11110?00010?1100000?110??010?111010???01100?1111

Elassoneuria_madeconeuria

1010?001110110?0???10?01111100011111101010001011010??011101111101011000?1111

Elassoneuria_elassoneuria

1010?0011101110100?10?01111100011011101010001111010??011101111101001010??111

Oligoneuriella_rhenana

0010?001110110?100?11001100?0001110110111000011101101110?111?11010011111?111

Oligoneuriella_pallida

0010?001110110?100?11101100?00011101101111000?110?101110?111011010011111?111

Oligoneuriella_orontoensis

0010??011101110110?11001100?0001110?10111?000?1???????????????1011011110?111

Oligoneuriopsis

1010?0011101110110?10?01100?0001110110111100111101101110?111?110110111101111

Oligoneurisca_borysthenica

1110?101?0?0010110?0??10000?110111111111110000??11101110?1110110100???0??111

Rianilaneuria_diminuta

?????????????????????????????????????????????00?11101110?11111100?01110?0111

;
