# Supplementary material for: Unmasking a gap: A new oligoneuriid fossil (Ephemeroptera: Insecta) from the Crato Formation (upper Aptian), Araripe Basin, NE Brazil, with comments on Colocrus McCafferty
Source: PLoS One. 2020 Oct 28;15(10):e0240365. doi: 10.1371/journal.pone.0240365 (PMC7592730; doi:10.1371/journal.pone.0240365)
Supplement: Attachment — Submitted filename: Response to Reviewers.docx [file pone.0240365.s003.docx]

Dear Dr. Martha Richter

We wish to submit the reviewed version of the research article titled “Unmasking a gap: a new oligoneuriid fossil (Ephemeroptera: Insecta) from the Crato Formation (upper Aptian), Araripe Basin, NE Brazil, with comments on *Colocrus* McCafferty”. Here we include a marked-up copy and an unmarked version of the revised manuscript.

The reviewers recommended Minor Revision. Concerning the considerations made by the Editor, we substituted Figure 1, that was made using GIS, since the previous map was modified from Barling et al. (2015).

Reviewer 1 recommended changes in the phylogeny image (Figure 3), such as the addition of a dagger symbol to indicate taxa known only by fossils and addition of support values to all branches, besides minor modifications of the text. We complied with all changes.

Concerning Reviewer 2, most of the suggestions were related to the phylogenetic analysis, such as to perform an exhaustive search instead of a traditional search and to calculate branch support using Relative Bremer. These modifications were made. Minor modifications of the text were also made after the reviewer’s suggestion.

Please see below all our comments to each reviewers’ suggestions.

We thank you and the reviewers for the comments made on our submission. We have addressed all of them.

Thank you for handling this manuscript.

Sincerely,

Arianny Storari

Reviewer #1

1) Recommend adding dagger symbol (†) to fossil taxa on the phylogeny.

Agreed. We added to the figure and the supplementary material.

2) Please add bootstrap values to the phylogeny – if numbers would clutter too much, perhaps consider a color scheme red/yellow/green for certain bootstrap ranges. At the moment, only a few support values are reported in the text.

Agreed. We added bootstrap values to the figure and the Methods section.

3) Line 20: “mayflies stand out” – why do they stand out? Morphologically conspicuous?

Accepted. We changed to “mayflies stand out in abundance".

4) Line 37: if you have quantitative information on the specimen-level disparity between larvae and adults from the McCafferty paper, could be worth including (ie 10:1 ratio, or similar)

Accepted. We included quantitative information on the adults and larval specimens’ proportions from McCafferty (1990), and also from a controlled excavation done at the Crato Formation by one of us (AAFS).

5) Line 47: “Indomalayan” is more commonly used in place of “Oriental Region”

Accepted.

6) Line 89: although not required, it’s often helpful in morphological matrices that include fossil taxa to report the number of missing character states. I recommend including the total % of missing cells in the matrix, the average number of cells per taxon, and the number of missing cells for fossils/new taxon. These quick stats will help readers assess how missing data may be informing relationships.

Accepted. We included the average percent of missing data of the extant and extinct OTUs.

7) Line 94: “nonaddictive” should be “non-additive” I believe

Accepted.

8) Line 389: unclear what “hendata” is

Agreed. "hendata format" was replaced to "tnt format".

Reviewer #2

1) Line 39. You could specify that the species refer to Ephemeroptera. As it is written it can be confused with Oligoneuriidae species;

Accepted.

2) Line 49. I strongly suggest you include the information "gender unknown";

Accepted, but we corrected from gender to genus.

3) Lines 73, 80, 223, 234. In original description of *Colocrus indivicum* (McCafferty 1990), the paratype code is: AMNH 43499. I believe that a kind of typo must have occurred;

Agreed.

4) Lines 85, 86. Just for reasons of order, I suggest that the information be inverted, mentioning character 32 first and then 54;

Accepted.

5) Line 89. 21 ingroup taxa (OTU) were used in Massariol et al. (2019), so you excluded from yours analyses *Lachlania aldinae*, *Lachlania* sp., and *Homoeoneuria* sp. Please explain the reason for this exclusion or make it clear that you have not used exactly all OTUs from Massariol et al. (2019);

We have excluded from the analysis *Lachlania* sp. and *Homoeoneuria* sp. because these are undetermined species whose genera were included in the analysis using known species. Since the aim of the analysis was to unravel the phylogenetic position of LPU 1696 within Oligoneuriidae, these exclusions do not compromise the results. *Lachlania aldinae* has now been included. We have incorporated such explanation at the Material and Methods section and corrected the number of analyzed ingroup taxa.

6) Line 94. Why didn't you do an exhaustive search (implicit enumaration command), since the matrix is small? I think it is worth doing this analysis, as this will explore the total number of possible trees for the data matrix;

Accepted. We have redone the analysis using the implicit enumeration command, and changed at the Material and Methods section. The number of suboptimal trees was the same.

7) Line 96. Please explain the choice of k value;

Accepted. We have explained in more detail.

8) Line 98. Why did you choose bootstrap to calculate branch support? For parsimony analyzes there are alternatives with less bias, such as Relative Bremer support (RB) or frequency difference (GC). For more details see: Goloboff PA, Farris JS, Kallersjo M, Oxelman B, Ramirez MJ, Szumik CA. 2003. Improvements to resampling measures of group support. Cladistics 19: 324–332;

Accepted. We have used both, as Reviewer 1 asked for Bootstrap values.

9) Line 100. Please specify "combined data". Later you explain that it is the matrix with larvae and adults characters, but I think it is better to make this information clear at "Material and Methods" topic;

Accepted.

10) Line 101. I counted 7 characters instead of 5: 27, 28, 30, 32, 33, 34 and 37. Please check it again;

Accepted.

11) Line 124. I suggest you include the values of "total fit" and "adjusted homoplasy";

Accepted.

12) Line 128. For a clearer figure caption, I suggest you include the information that characters are from larvae and adults;

Accepted.

13) Line 129. Not all synapomorphies were depicted in Fig. 4. Explain which ones have been suppressed and why. In addition, I did the analysis again in TNT and at Oligoneuriidae clade, another synapomorphy was recovered (in addition to the ones you found): 14 (0). Review the analysis;

We have omitted synapomorphies other than those that support the monophyly of Oligoneuriidae and Oligoneuriinae+Incogemininae for clarity. This is now explained in the figure legend. Also, we have reviewed the analysis and, indeed, the character 14 was overlooked by us. We have now included in the text and in Figure 4.

14) Please check if there are 5 or 6 (see comment 13);

Accepted.

15) Lines 133-144. If you prefer, you can simplify by “8:1”, “10:1” and so on instead of using “character 8: state 1” and “character 10: state 1”;

Accepted.

16) Line 146. "Inconclusive" is a vague term, so I suggest you explain the results obtained. Did *Colocrus* go into polytomy? Was there no high support from the branches?;

Accepted. We have added a more detailed explanation in the text.

17) Line 157. In the sentence did you really mean *Oligoneuria* and not Oligoneurinae?

We really meant *Oligoneuria*, as it is the OTU of our analysis and the type genus of Oligoneurinae.

18) Lines 191, 192. I also suggest you pointing out the CuA1 and CuA2 veins in figure 5B;

Accepted.

19) Lines 282, 295. Did you mean the divergence between Oligoneuriinae and Incogemininae or the divergence between Oligoneuriinae + Incogemininae and other Oligoneuriidae? If it is the first option, replace "+" with "-", so the idea will be clearer.

Accepted. We have changed “+” to “and”.

20) Line 283. The current distribution of *Chromarcys* is explained both by dispersion, at ancient moment, and by vicariance later. Review this;

Accepted.

21) Line 284. Did you mean fossil or extant specimens? Please clarify this information.

Accepted. We included "fossilized specimens".
